# Supplementary material for: Structural Plasticity and Ligand Promiscuity of CYP3A4 Revealed by Cryo-EM
Source: bioRxiv. 2026 Sep 21:2026.09.01.748687. Preprint. [Version 5] doi: 10.64898/2026.09.01.748687 (PMC13622003; doi:10.64898/2026.09.01.748687)
Supplement: Supplementary file 1 [file NIHPP2026.09.01.748687V5-supplement-1.pdf]

bioRxiv preprint doi: https://doi.org/10.64898/2026.09.01.748687; this version posted September 21, 2026. The copyright holder for this preprint (which was not certified by peer review) is the author/funder, who has granted bioRxiv a license to display the preprint in perpetuity. It is made available under a CC-BY 4.0 International license.

Supplementary Information

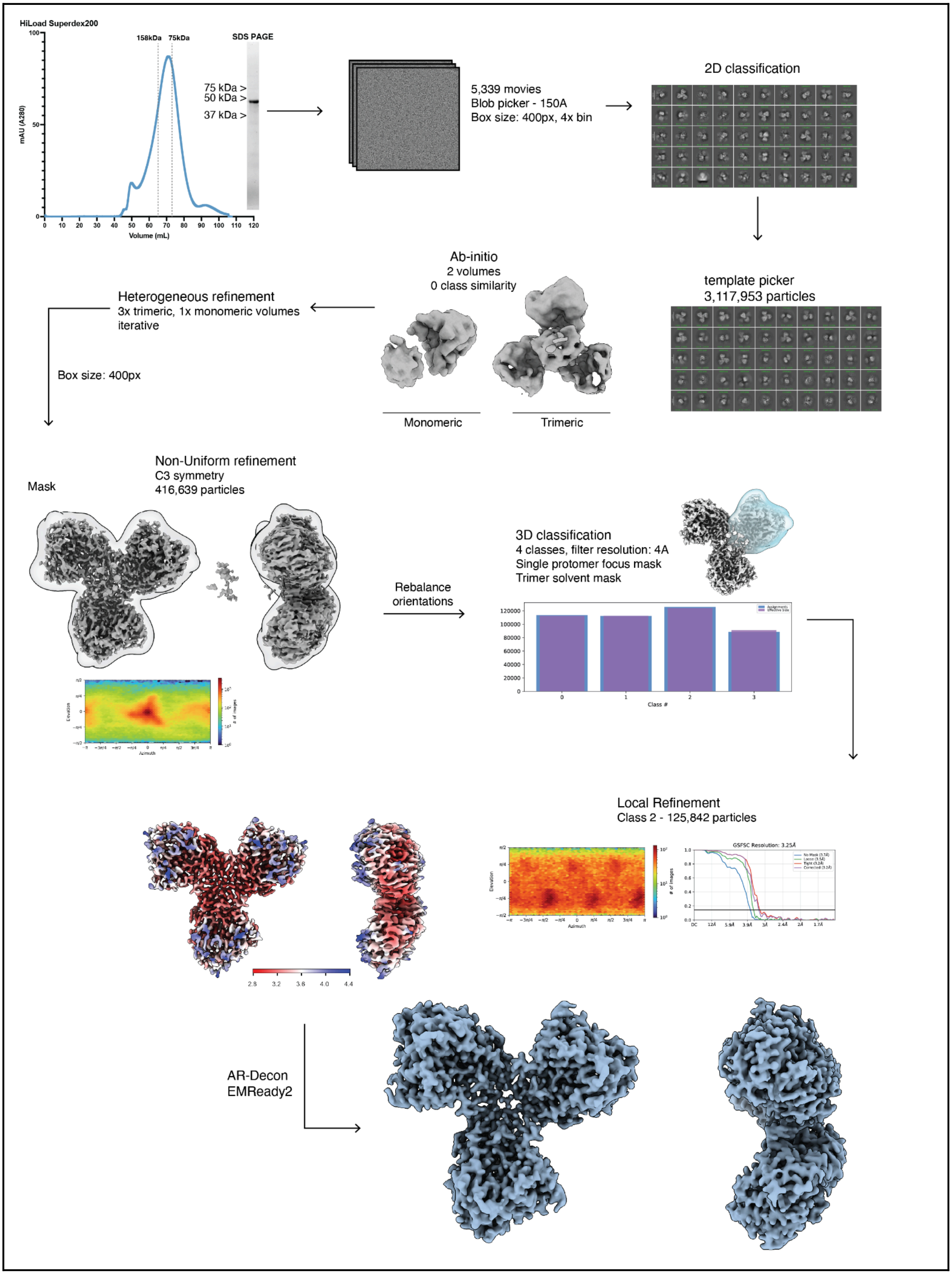

bioRxiv preprint doi: https://doi.org/10.64898/2026.09.01.748687; this version posted September 21, 2026. The copyright holder for this preprint (which was not certified by peer review) is the author/funder, who has granted bioRxiv a license to display the preprint in perpetuity. It is made available under a CC-BY 4.0 International license.

Supplementary Figure1: Cryo-EM processing pipeline of CYP3A4. Particles were picked by blob picker followed by 2D classification through cryoSPARC Live. Selected particles were used to generate 2 Ab-initio models which resulted in one volume representing the trimeric state, and a monomeric state used as decoy. After iterative rounds of heterogeneous refinement, 3D classification into 4 volumes sorted the highest resolution into Class 2. The particles were then used for Non-Uniform refinement which resulted in a 3.25 Å map. The final map was sharpened using EMReady2.

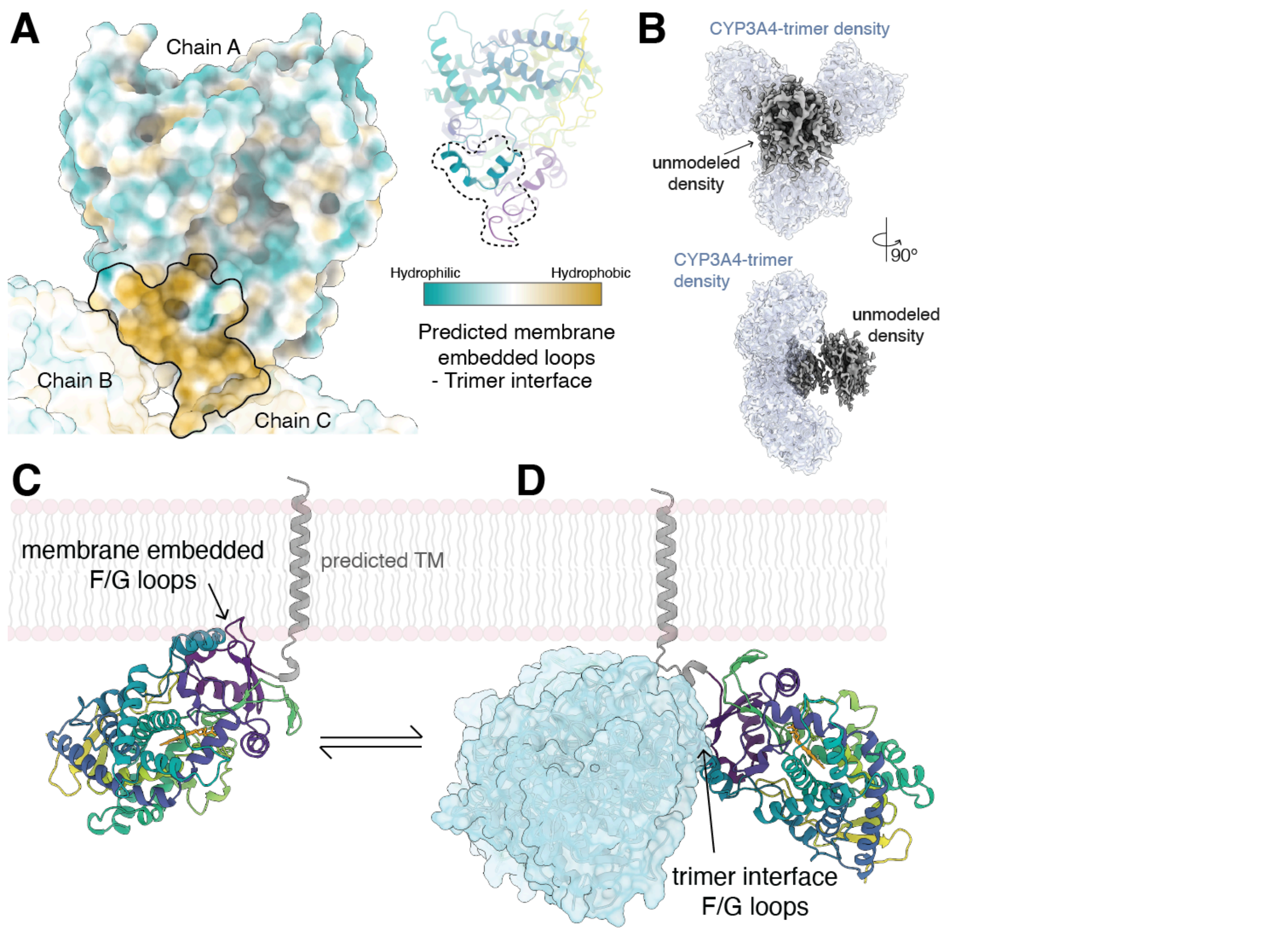

Supplementary Figure2: **A**. Surface of a CYP3A4 protomer (left), colored from hydrophilic (teal) to hydrophobic (yellow) by the Kyte-Doolittle scale. The region outlined in black corresponds to the trimer interface, which is predominantly hydrophobic and coincides with the predicted membrane-embedded surface and the trimer interface observed in this study. The corresponding secondary structure is shown at right (viridis), with dashed circles indicating the hydrophobic patch highlighted in the left panel.
B. Lower contour map of the unliganded CYP3A4, representative of all of the maps in this study, showing unmodeled density (gray). **C**. Monomeric CYP3A4 (shown in the viridis color scheme) in a predicted membrane-embedded configuration, with the F/G loops inserted into the lipid bilayer as predicted by MD (Spanke et al., 2025). The predicted transmembrane helix is shown in gray. **D**. Cryo-EM trimeric assembly, in which the G-helix composes the trimer interface rather than engaging the membrane. We omit the predicted transmembrane helices for the two copies of CYP3A4 represented in cyan surface view. The double arrow indicates the equilibrium between these two states.

bioRxiv preprint doi: https://doi.org/10.64898/2026.09.01.748687; this version posted September 21, 2026. The copyright holder for this preprint (which was not certified by peer review) is the author/funder, who has granted bioRxiv a license to display the preprint in perpetuity. It is made available under a CC-BY 4.0 International license.

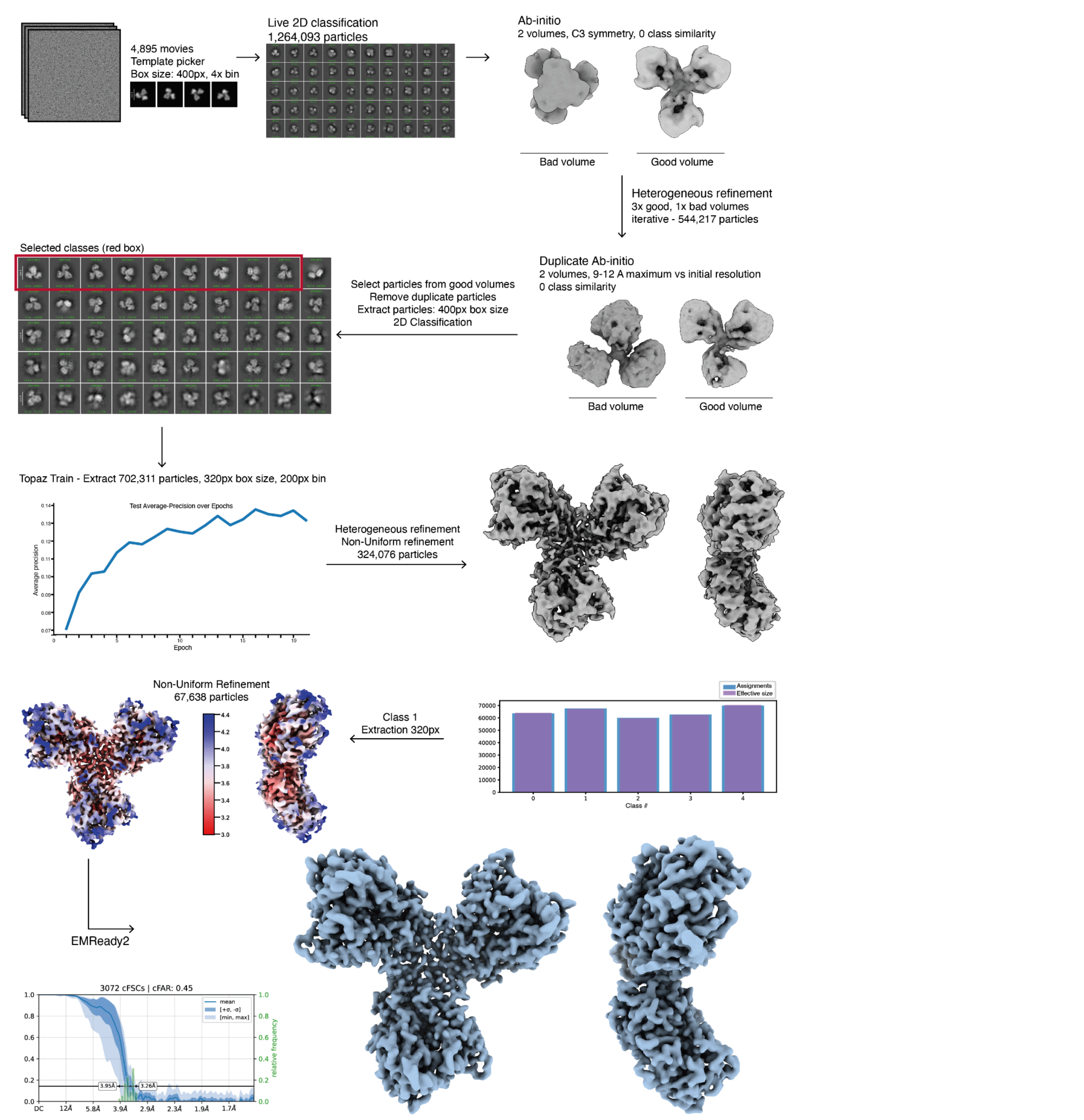

Supplementary Figure3: Cryo-EM processing pipeline of ritonavir-bound CYP3A4. Particles were picked using templates from the unbound dataset followed by 2D classification through cryoSPARC Live. Selected particles were used to generate 2 Ab-initio models for heterogeneous refinement. After iterative rounds of refinement, 2D classification was used to select a subset of views for Topaz training. The Topaz picked particles were further refined through heterogeneous refinement. A last round of 3D classification into 4 volumes led to one class with a final reported resolution of 3.55 Å at an FSC of 0.143. The final map was sharpened using EMReady2.

bioRxiv preprint doi: https://doi.org/10.64898/2026.09.01.748687; this version posted September 21, 2026. The copyright holder for this preprint (which was not certified by peer review) is the author/funder, who has granted bioRxiv a license to display the preprint in perpetuity. It is made available under a CC-BY 4.0 International license.

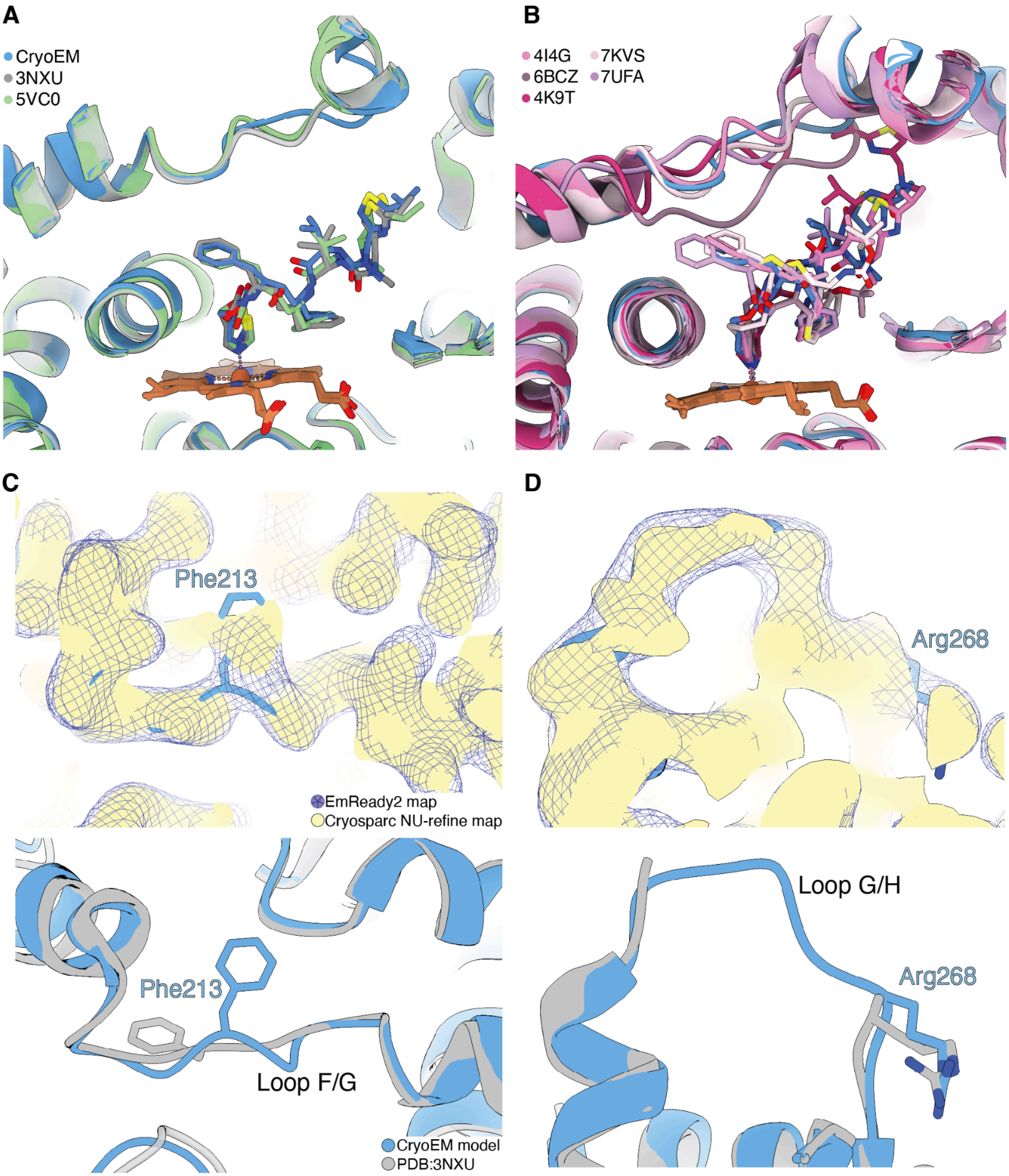

Supplementary Figure4: **A.** Overlay of CYP3A4-ritonavir structures PDB: 3NXU (gray), 5VC0 (green) and the cryo-EM model in this study (blue). **B.** Overlay of various ritonavir analogues from diverse publications (shades of pink, PDB: 4I4G, 6BCZ, 4K9T, 7KVS, 7UFA) showing the dynamics of loop F/G in the presence of a highly coordinated ligand like ritonavir and ritonavir-derived molecules. These molecules all share a coordination to the heme iron through a nitrogen-containing heterocyclic ring. **C.** Densities of the F/G loop

bioRxiv preprint doi: https://doi.org/10.64898/2026.09.01.748687; this version posted September 21, 2026. The copyright holder for this preprint (which was not certified by peer review) is the author/funder, who has granted bioRxiv a license to display the preprint in perpetuity. It is made available under a CC-BY 4.0 International license.

highlighting residue Phe213. The Non-Uniform refined map from cryoSPARC is shown in yellow, overlaid against the EMReady2 map (mesh) derived from the cryoSPARC map. EMReady2 shows a connected density for the loop along with side chain density for Phe213. This residue is solvent exposed in the cryo-EM model, and coordinates the ligand in the crystal structure PDB:3NXU (bottom panel). **D.** As in **C**, EMReady2 map shows a connected density to residue Arg268, in agreement with the rotameric state in the crystal structure (bottom panel). The loop preceding this residue is unmodeled in PDB:3NXU.

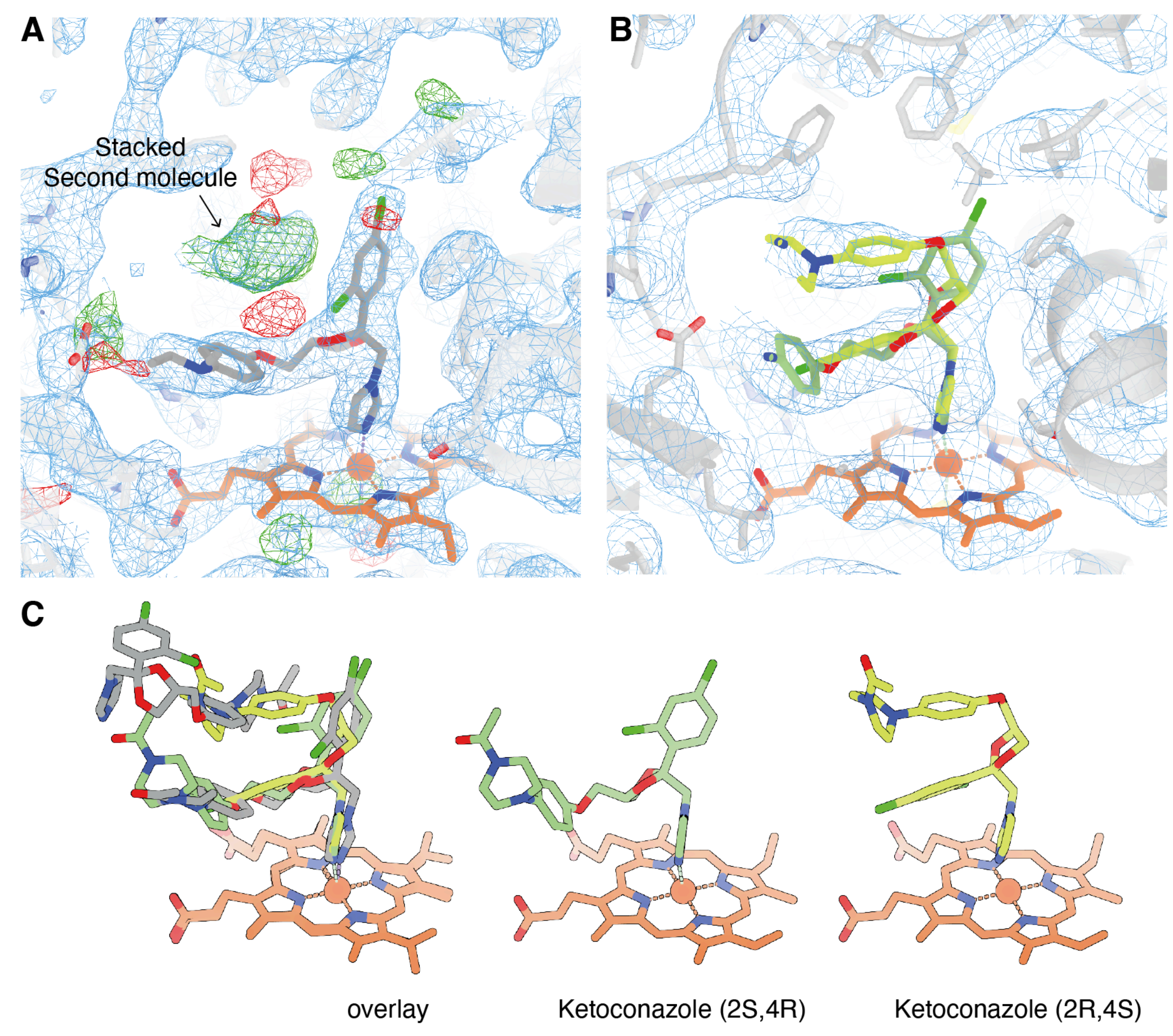

Supplementary Figure5: **A.** Re-refined crystallographic map showing difference density (green) for a second stacked ketoconazole molecule, without any connecting density. **B.** cryo-EM density map and model of ketoconazole in two mutually exclusive states with a continuous density between the two molecules. **C.** Atomic view of the ketoconazole molecules coordinating the heme-iron. Left shows an overlay between the crystal (gray) and EM (green) structures. Middle shows the (2S,4R) isomer, right shows the (2R,4S) isomer.

bioRxiv preprint doi: https://doi.org/10.64898/2026.09.01.748687; this version posted September 21, 2026. The copyright holder for this preprint (which was not certified by peer review) is the author/funder, who has granted bioRxiv a license to display the preprint in perpetuity. It is made available under a CC-BY 4.0 International license.

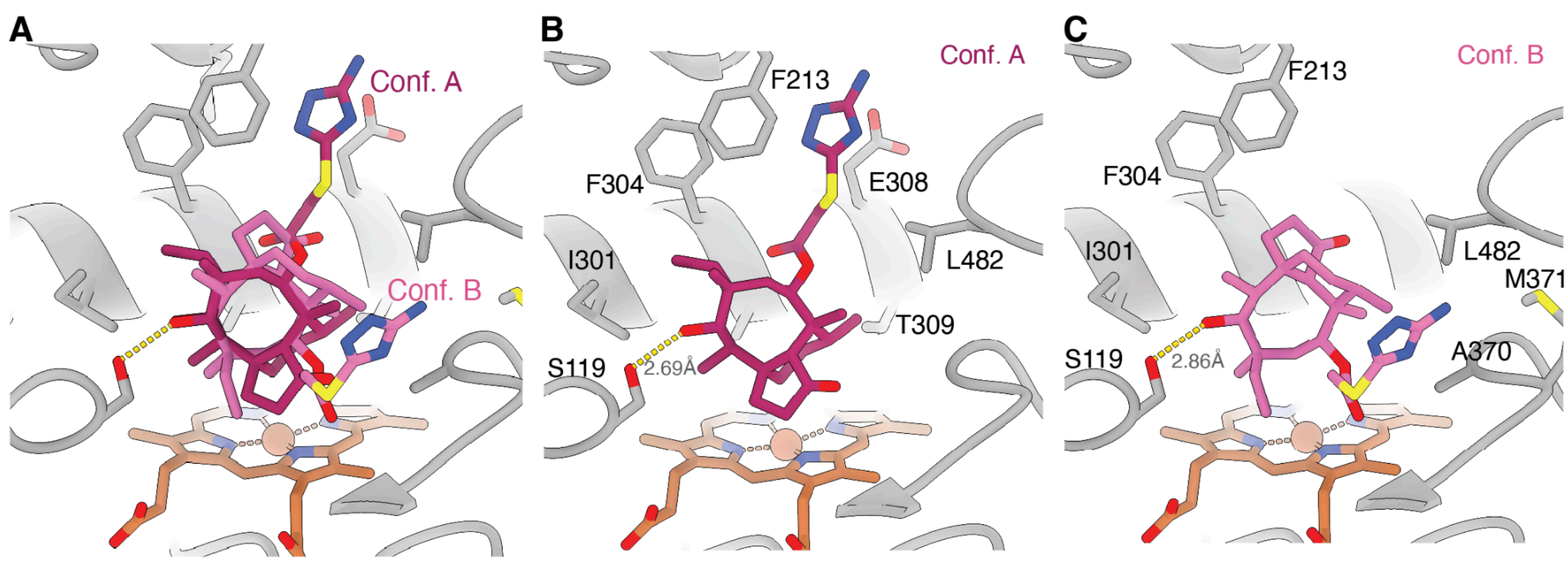

Supplementary Figure6: **A.** Conformations of azamulin observed in this study colored in dark (Conf. A) and light (Conf. B) shades of pink. **B-C.** Conformation A and B, respectively, along with the side chain contacts to the CYP3A4 active site.

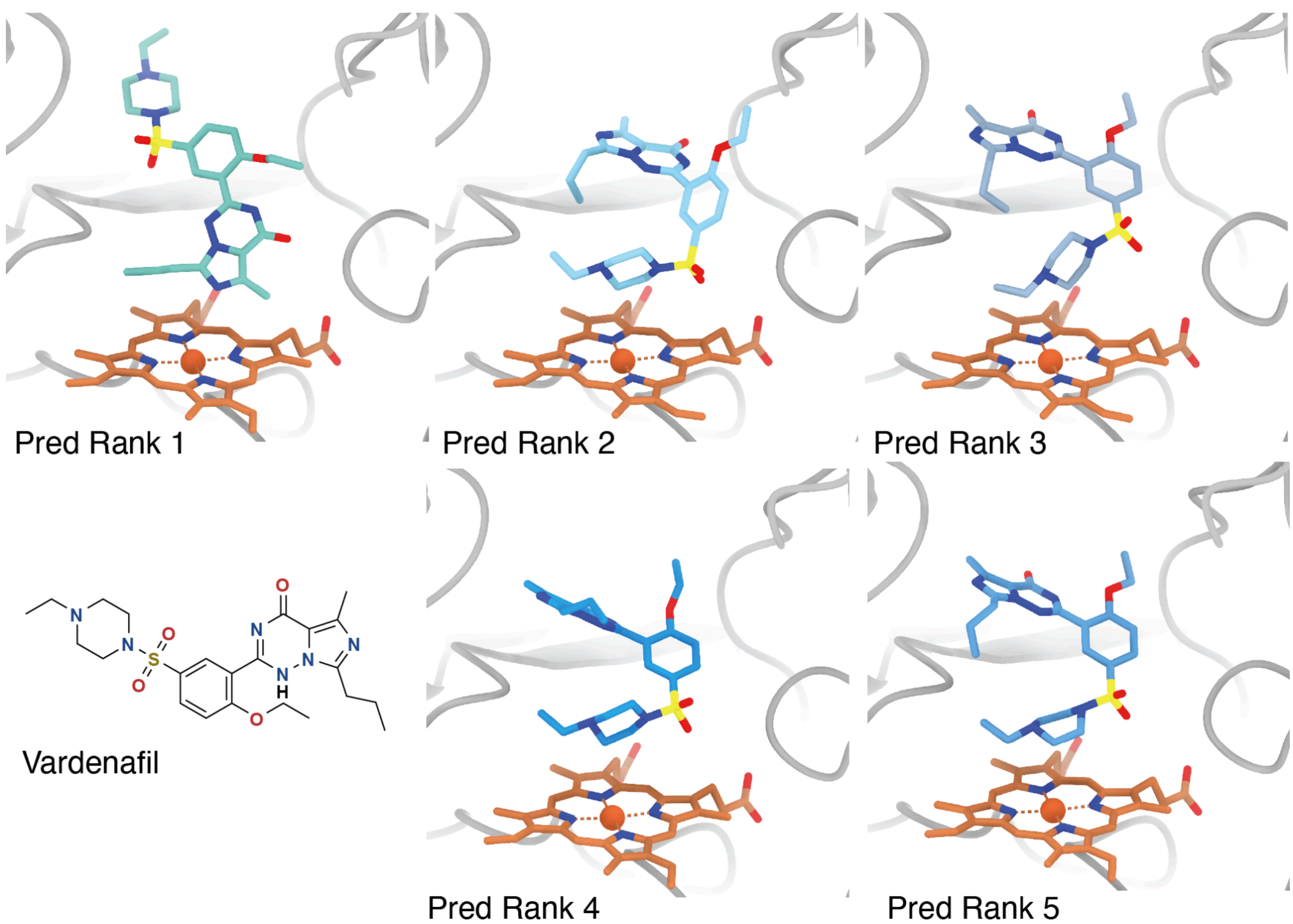

Supplementary Figure7: Ranked Boltz-2 predictions of vardenafil-bound CYP3A4. Vardenafil is shown in shades of blue, heme in orange. The chemical structure of vardenafil is shown in the bottom left panel.

bioRxiv preprint doi: https://doi.org/10.64898/2026.09.01.748687; this version posted September 21, 2026. The copyright holder for this preprint (which was not certified by peer review) is the author/funder, who has granted bioRxiv a license to display the preprint in perpetuity. It is made available under a CC-BY 4.0 International license.

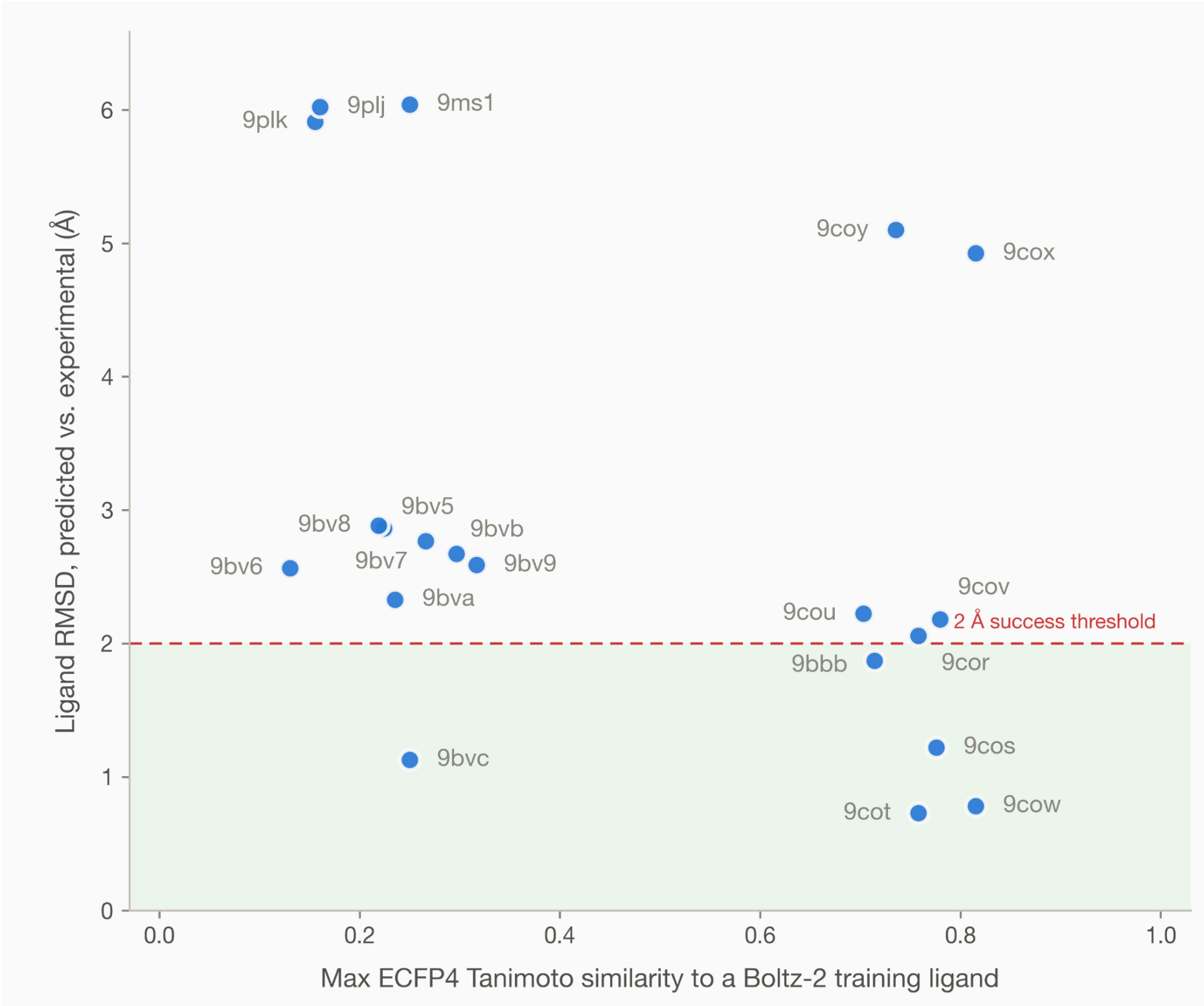

Supplementary Figure8: For each held-out structure, maximum ECFP4 Tanimoto similarity to the most similar Boltz-2 training ligand, plotted against ligand RMSD between predicted and experimental pose. The red dashed line marks the 2 Å success threshold.
